# A combination of computational and experimental approaches identifies DNA sequence constraints associated with target site binding specificity of the transcription factor CSL

**DOI:** 10.1093/nar/gku730

**Published:** 2014-08-11

**Authors:** Rubben Torella, Jinghua Li, Eddie Kinrade, Gustavo Cerda-Moya, Ashley N. Contreras, Robert Foy, Robert Stojnic, Robert C. Glen, Rhett A. Kovall, Boris Adryan, Sarah J. Bray

**Affiliations:** 1 Department of Chemistry, University of Cambridge, Cambridge, UK; 2 Department of Physiology, Development and Neuroscience, University of Cambridge, Cambridge, UK; 3 Department of Genetics, University of Cambridge, Cambridge, UK; 4 Cambridge Systems Biology Centre, University of Cambridge, Cambridge, UK; 5 University of Cincinnati College of Medicine, Department of Molecular Genetics, Biochemistry and Microbiology, 231 Albert Sabin Way CARE4836, Cincinnati, OH 45267-0524, USA

## Abstract

Regulation of transcription is fundamental to development and physiology, and occurs through binding of transcription factors to specific DNA sequences in the genome. CSL (CBF1/Suppressor of Hairless/LAG-1), a core component of the Notch signaling pathway, is one such transcription factor that acts in concert with co-activators or co-repressors to control the activity of associated target genes. One fundamental question is how CSL can recognize and select among different DNA sequences available *in vivo* and whether variations between selected sequences can influence its function. We have therefore investigated CSL–DNA recognition using computational approaches to analyze the energetics of CSL bound to different DNAs and tested the *in silico* predictions with *in vitro* and *in vivo* assays. Our results reveal novel aspects of CSL binding that may help explain the range of binding observed *in vivo*. In addition, using molecular dynamics simulations, we show that domain–domain correlations within CSL differ significantly depending on the DNA sequence bound, suggesting that different DNA sequences may directly influence CSL function. Taken together, our results, based on computational chemistry approaches, provide valuable insights into transcription factor-DNA binding, in this particular case increasing our understanding of CSL–DNA interactions and how these may impact on its transcriptional control.

## INTRODUCTION

Transcription is controlled through a number of mechanisms including the specific interactions between DNA and regulatory proteins. Sequence-specific protein–DNA recognition (1–3) occurs through both direct (base) and indirect (shape) readout of a DNA sequence by transcription factors (TFs). Base readout involves the recognition of base-specific groups (principally through hydrogen bond donor/acceptor and hydrophobic interactions) by complementary groups present on amino acid side chains in the targeting protein (4). Shape readout describes base pairs that are not directly in contact with the protein, but likely influence the DNA structure and shape and hence the protein–DNA interactions (5). This combination results in each TF having a specific repertoire of DNA sequences with which it can form complexes of varying stabilities. While considerable progress has been made in understanding the basic principles of protein–DNA recognition, it is often unclear what spectrum of binding sites *in vivo* is functionally relevant for a given TF. Furthermore, the consequences of different DNA target sites on the (dynamic) behavior of the bound protein, and hence the functional outcome of binding, are rarely considered.

The TF CSL provides a powerful and important model to investigate the relevance of protein dynamics in DNA bound complexes. CSL is the nuclear effector of the Notch signaling pathway (6). In un-stimulated cells CSL binds DNA in the presence of a co-repressor, blocking transcription. The interaction of the Notch receptor with its ligands initiates a cascade of cleavage reactions that culminate in the release of the intracellular domain of the Notch receptor, NICD (7–9). This fragment binds directly to CSL, recruiting the co-activator Mastermind (MAM) (10–12) to promote active transcription (13,14). Thus, the protein–protein interactions CSL makes with different binding partners are critical in determining the regulatory outcome of DNA binding.

Structural studies have illuminated how CSL interacts with both co-regulators and DNA. Its conserved core consists of three domains: the N-terminal domain (NTD), the β-trefoil domain (BTD) and the C-terminal domain (CTD) (15). NTD and BTD both contribute to DNA recognition, interacting with base pairs in the major and minor grooves of the DNA helix, respectively. The CTD binds the ankyrin-repeat (ANK) domain of NICD and, in the tertiary complex, both CTD and NTD contact MAM through a long α-helix (10,16). High affinity binding of the so-called RAM region (RBP-Jk-associated-molecule) of NICD to BTD is also important for recruiting NICD to CSL and in facilitating the binding of MAM (17). Finally, CTD and BTD also participate in binding to co-repressors (18). This high-resolution picture provides a powerful starting point for molecular simulation studies to explore the binding space and its effects on the molecular dynamics (MD) of CSL.

As for many TFs, there already exists considerable information about DNA motifs that can be recognized by CSL. However, much of this derives from sequences that are evolutionarily related to the Hairy-Enhancer of split (HES) target genes, which may distort the position weight matrix (PWM) generated for CSL. Indeed, such PWMs cannot fully account for all of the sites occupied *in vivo* by CSL, based on genome-wide chromatin immunoprecipitation studies. Additionally, a recent *in vitro* analysis (19) has shown that CSL may bind a large repertoire of DNA sequences. Therefore, one question is whether the physico-chemical properties evident in the structure of CSL can be used for predicting the range of plausible DNA binding motifs. A second question is whether different DNA sequences bound by CSL can influence its inter-domain dynamics, thereby potentially impacting CSL function.

One approach to investigate TF-target recognition and specificity is to apply computational methods that model the chemical interactions between the protein and DNA, using information from the crystal structure. Here we have applied this strategy to CSL, performing an *in silico* mutational analysis of the binding motif and modeling the dynamics of the protein when it is bound to different DNA motifs. The results highlight two features of CSL sequence recognition that could not be inferred by sequence-based bioinformatics, and whose relevance we have demonstrated using biochemical and transcriptional assays. Furthermore, the simulations show that CSL responds differently depending on the precise sequence of the DNA: large differences between the domain–domain correlation pathways occur when CSL binds a canonical DNA binding motif CGTGGGAA compared to a lower affinity motif (CGTGTGAC) motif or to the unbound state. Such correlations could influence the recruitment of other proteins, including NICD, MAM and co-repressors. An important observation is that DNA may influence protein-binding events remote from the DNA-binding domain, by changing the dynamic regime of the complex.

## MATERIALS AND METHODS

### Preparation of the starting structure

The starting structure for the analysis was mouse CSL bound to DNA (HES-1 (*hairy and enhancer of split-1*) site 5’-TGTGGGAA-3’; PDB code 3BRG, resolution 2.2 Å), which contains all of the CSL conserved features but lacks the variable N-terminal residues (52 residues in mouse). The structure was checked for missing residues using Protein Preparation Wizard (Schrodinger, MAESTRO package, version 9.0). Two missing gaps (K197-L200, E255-T262, using the crystallographic numbering) were modeled using MODELLER (20,21) (version 9.8), aligning the primary sequence with the missing loops. The pKa's of the residues of the protein were calculated using the PROPKA (22) and H++ (23) software. Residues were assigned a protonation state consistent with physiological pH (7.4). Five hundred iterations of molecular mechanics were performed and the lowest energy structure was taken as a starting point for further calculations.

Changes to the DNA consensus sequences were performed using the FOLDX software (24,25), a tool for energy calculations and macromolecular design. The consensus complex was energy minimized and the selected mutations in the DNA sequence were performed, adapting the conformations of the side chains of the residues interacting with the DNA fragment to a new low energy state in the presence of the base pairs of the new fragment. The relative binding energy result for each DNA sequence has been also normalized taking into consideration possible deformations that may have occurred on the protein and on the DNA. Logos for MotifMap/TRANSFAC and FOLDX sequences were created using Weblogo (26).

### MD simulation protocol

MD simulations were performed with the AMBER11 package (http://ambermd.org/) (27), using the AMBER FF99SB force field (28). All calculations were made with the CUDA enabled version of PMEMD (29), using TESLA GPUs at the High Performance Computing cluster (University of Cambridge). Four TESLA-GPU's perform ca. 4 ns/day, when computing a system of ca. 67 500 atoms. A dodecahedral box of water (TIP3P (30)) was built around the complexes and a physiological concentration of 0.15 M of NaCl was added to the box using the following equation: *N*(ions) = *N*(water molecules) × 0.15/55.555. A 1 nm non-bonded cutoff was used for the van der Waals interactions, while the Particle Mesh Ewald summation method was used to deal with long-range Coulomb interactions. The Berendsen thermostat was used to control temperature and pressure (31).

The following protocol was used for all the simulations: (i) *in vacuo* minimization (1000 steps); (ii) minimization, keeping the complexes fixed, allowing water molecules and ions to equilibrate (2000 steps); (iii) minimization of all the system, without restrictions (2000 steps); (iv) equilibration, 1 ns; (v) production phase of 48 ns. To improve the sampling of the MD simulation, three simulation replicas of 48 ns were performed with different starting velocities, for an overall simulation time of 720 ns. For the analysis, the first 8 ns of each trajectory were eliminated (as the system is equilibrating) and the following 40 ns of simulation from each trajectory were concatenated into a macro-trajectory of 120 ns for each system.

All the analyses were performed with packages from the AMBERTOOLS 1.4 and GROMACS package, after the trajectories were transformed into a suitable format. Root Mean Square Deviation (RMSD) and Root Mean Square Fluctuation (RMSF) of the MD trajectories have been performed as in (32–34). The annotation of protein residues and DNA bases follows the order of residues and DNA specified in the original PDB file; the first residue is the 53rd residue of the original structure. The unbound structure of the protein was created from the 3BRG crystallographic structure of the protein bound to DNA, by stripping DNA from the complex followed by optimization (energy minimization) in implicit solvent. After this step, the unbound structure followed the protocols described previously for the other structures.

### Internal coordination and rigidity

In order to provide a simple and sequence-related one-dimensional descriptor of the contribution of each residue contributing toward the connectivity/cooperativity of the motion within the protein, an analysis based upon signal propagation was used (35). In order to describe the correlation between atom pairs undergoing dynamics, a matrix ICRM (Internal Coordination and Rigidity Matrix) was used for protein–ligand (36,37) and protein–DNA interaction (32). A threshold distance of 30 Å was considered, keeping in consideration the distance between the residues that interact with DNA and the residues in the CTD domain.

### *In vitro* binding experiments (electro-mobility shift assay)

GST-Su(H) [110–594] (38) fusion protein was purified from 500 ml transformed *Escherichia coli* strain BL21 cells using Glutathione-Agarose (Sigma-Aldrich) and concentrated, by centrifuging at room temperature for 10 min, using Amicon^®^ Ultra-0.5 Centrifugal Filter Units (Millipore) to ∼1 mg/ml. The oligonucleotide 5′-ACCGAAACCGTGGGAACTGGTAGAAAG-3′ and its reverse complement, 5′-CTTTCTACCAGTTCCCACGGTTTCGGT-3′, were labeled using the biotin 3′-end labeling kit following the manufacturer's instructions (Pierce). The two single-stranded oligonucleotides were then annealed. The DNA-binding reactions contained 1 μl of GST-Su(H) and 25 fmol of biotin labeled DNA in a 5 μl volume binding reaction (150 mM KCl, 50 mM Tris pH 7.4, 1 mM DTT, 2% polyvinyl alcohol). In total 30 ng/μl of poly(dI·dC) was also included as a non-specific competitor. Different amount of unlabeled double-stranded oligonucleotides (Supplementary Table S1) were added as specific competitors. Binding reactions were incubated on ice for 30 min. Oligonucleotide–protein complexes were then separated on 5% native polyacrylamide gels at 75V in 0.5× TBE buffer. The products were then transferred to a Biodyne B membrane (Pierce) at 30V in 0.5× TBE buffer followed by UV cross-linking. The biotin-labeled reaction products were then visualized by incubation with streptavidin horseradish peroxidase conjugate and subsequent incubation with ECL chemiluminescent reagents.

### Luciferase assays

Oligonucleotides containing the indicated CSL motifs (Supplementary Table S2) were used to generate fragments for cloning into a luciferase vector containing a minimal promoter from the hsp70 gene (pGL3::Min(39)) (Supplementary Table S3). *Drosophila* S2 cell culture and transfections were as described previously (39) and expression levels were compared in the presence and absence of pMT-NICD after 16–20 h induction with 0.5 mM CuSO_4_. Empty pMT was used as a control and the fold change was calculated as a ratio between values obtained with pMT-NICD and empty pMT. As the reporters also contained binding sites for Grainyhead (Grh), (40), to analyze repression cells were co-transfected with pMT-Grh in place of pMT-NICD in combination with the indicated luciferase reporter construct. For assays investigating CSL mutants, the transfections contained the indicated pMT-CSL constructs in combination with pMT-NICD. Controls with pMT-NICD or pMT-CSLwt were combined with empty pMT in the appropriate ratios. At least three biological replicates were performed for all experiments.

### AAA mutants and *in vivo* rescue assays

The choice of residues for mutagenesis involved taking into consideration different factors: the number of communication pathways, structural factors and interactions with other macromolecules. Three residues have been selected for mutation to alanine: T365, F366 and Y367, based on the fact that they are involved in domain–domain communication and are not involved in interactions with other proteins/DNA or are structurally relevant for CSL. For the modeling experiments, mutations were introduced into the CSL structure, bound to the consensus TGTGGGAA DNA sequence (PDB ID: 3BRG), using MODELLER 9v8. The same protocol described before was then used for analyzing the 413AAA mutant.

For the *in vivo* experiments, a CSL genome rescue construct was produced by amplifying the CSL locus delineated by the neighboring genes lethal(2)35Bg (NM_080199.2) and crinkled (NM_165099.2) (see Supplementary Table S3). The ∼6.3 kb of *Drosophila* CSL locus was then cloned into a pAttB plasmid (41) to generate CSLwt. In order to generate the genomic rescue mutant CSL413AAA, site-directed mutagenesis was performed to replace the residues Q413, F414 and Y415 with AAA (Supplementary Table S4). The same mutagenesis was performed to generate the AAA mutated version in pMT for luciferase assays.

*Drosophila* transgenic lines were generated for both CSLwt and CSL413AAA by inserting the genomic rescue construct into Φ86Fb located on the third chromosome using ΦC31 integrase-mediated system (41,42). Chromosomes carrying the genomic rescue constructs were then crossed into the *Su(H)[SF8]* (43) lethal allele for *Drosophila* CSL. Rescue experiments were performed by crossing *Su(H)[SF8]*/*CyO*; *CSLwt*/*CSLwt* or *Su(H)[SF8]*/*CyO*: *CSL[413AAA]*/*CSL[413AAA]* to *Su(H)[AR9]*/*CyO* (43) and scoring the percentage of viable progeny without the *CyO* marker.

### Isothermal titration calorimetry of CSL–DNA complexes

The cloning, expression and purification of *Mus musculus* CSL (amino acids 53–474) was described previously (17). Briefly, CSL was overexpressed as a GST-fusion protein in bacteria and isolated from crude lysate by affinity chromatography. After cleaving off the fusion tag, CSL was purified to homogeneity using a combination of ion exchange and size exclusion chromatography.

Oligonucleotides were ordered from Eurofins MWG Operon. Each single-stranded DNA was resuspended in water and purified over a GE Healthcare Life Sciences Resource Q ion exchange column. The resulting peak was pooled, concentrated and exchanged into a buffer of 10 mM Tris (pH 7.6), 500 mM NaCl and 1 mM MgCl_2_ in an Amicon Ultra centrifugal filter (3000 MWCO). Concentrated single-stranded DNAs were spectroscopically quantified at 260 nm, combined in equimolar amounts, boiled for 10 min and allowed to cool to room temperature to ensure optimal duplex annealing.

Purified components to be used in isothermal titration calorimetry (ITC) experiments were degassed and buffer-matched by running over a size exclusion column into the experimental buffer of 50 mM sodium phosphate (pH 6.5) and 150 mM sodium chloride. Concentrations of the components were determined by UV absorbance at 260 nm (DNA) and 280 nm (CSL protein). All experiments were performed with a MicroCal VP-ITC microcalorimeter at 10°C using 10 μM macromolecule (CSL protein) in the cell and 100 μM ligand (DNA) in the syringe. Data was analyzed using the ORIGIN software and fitted to a one-site binding model. The reported binding data is the average of at least three individual experiments (*n* = 3).

## RESULTS

### Prediction of CSL binding repertoire from structural calculations using FOLDX

**Figure 1.**
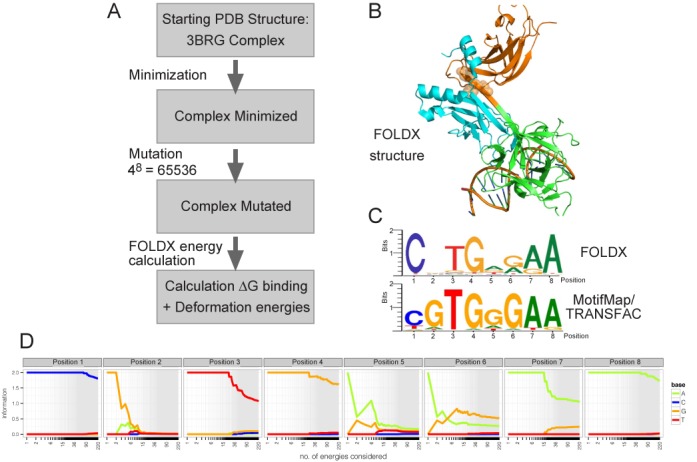
Overview of the FOLDX strategy and results. (**A**) Flow chart summarizing the FOLDX computational strategy. (**B**) CSL–DNA structure used for the analysis (CSL domains NTD cyan; BTD green, CTD orange). The position of the residues that were mutated to perturb inter-domain communication (see Figure 5) are indicated by orange spheres. (**C**) Comparison of information logos obtained from the >3 kcal/mol FOLDX predictions, weighted according to a Boltzmann distribution of energies, and from empirical binding analysis (RBPJ M01112 MotifMap/Transfac).

While there is substantial evidence that the DNA consensus sequences recognized with highest affinity by CSL are [T/C]GTG[G/T]GAA, these are insufficient to explain the full repertoire of *in vivo* binding events. For example, *in vitro* CSL can bind motifs with any nucleotide in position 5 with nanomolar binding affinity (44). Furthermore CSL appears to accept a broader range of nucleotides than the consensus guanine and adenine at positions 2 and 8 (45). In addition, many of the sites occupied *in vivo* do not conform to this strict consensus. We therefore set out to investigate whether protein structure-based *in silico* approaches could be used to provide information about the full repertoire of binding sites. To achieve this, the FOLDX software (24) was used to calculate changes in binding energy when varying the nucleotides within the constraints of the CSL–DNA co-crystal structure (Figure 1A). While energy minimization strategies on protein force fields were initially used to calculate the energy function for small molecules bound to proteins (46), FOLDX has been used to study the impact of mutations on protein stability and has subsequently been refined to encompass the possibility of studying protein–protein and protein–DNA interactions (25). The success of such an approach was shown for high-affinity binding sites of Pax6 (47), but has not been explored more widely and in particular its ability to detect lower affinity sites has not been assessed.

Starting from the X-ray structure where CSL binds its highest-affinity DNA motif comprised of eight nucleotides CGTGGGAA (PDB code 3BRG (17), Figure 1B), all 4^8^ permutations of an 8-nt motif were tested and the resulting 65 536 relative binding energies calculated (Figure 1A). After ranking these, a threshold of 3 kcal/mol, computed by the difference from the top predicted DNA sequence, was used as an initial cutoff, separating 220 putative ‘bound’ motifs from the residual 65 316 DNA sequences. These 220 ‘bound’ sequences were used to generate a binding logo, where the sequences were weighted according to their computed energies (Figure 1C). A similar logo was generated when the sequences were compiled without the weighting scheme (Supplementary Figure S1A) and neither logo was substantially altered by extending the number of sequences included (see Supplementary Figure S1B and the explorative tool at http://webtools.sysbiol.cam.ac.uk/MotifTool/ for further assessment of CSL FOLDX predictions and how different free energy cutoffs vary the resulting energy logo). Finally to assess whether interaction effects between any of the positions in the motif were detected, the conditional probability of finding a nucleotide at a certain position when a given nucleotide was present at another position was calculated (Supplementary Figure S1C). Although some specific interactions were detected, these were only found for nucleotides with a low likelihood of being present in the bound sequences, such that the overall probability for interactions was small.

Several striking features are evident when comparing the FOLDX binding logo with the energy logo obtained from empirical binding analysis (Figure 1C;^48^,^49^). First, FOLDX indicates a strong preference for a cytosine at position 1. Second, while there is thought to be a strong preference for guanine at positions 2 and 6, FOLDX indicated much greater sequence tolerance at these positions with little preference at position 2 and tolerance for G or A at position 6. Finally, FOLDX results also suggest that considerable variability could be accepted at position 5, where there is conventionally thought to be a preference for G or A.

**Figure 2.**
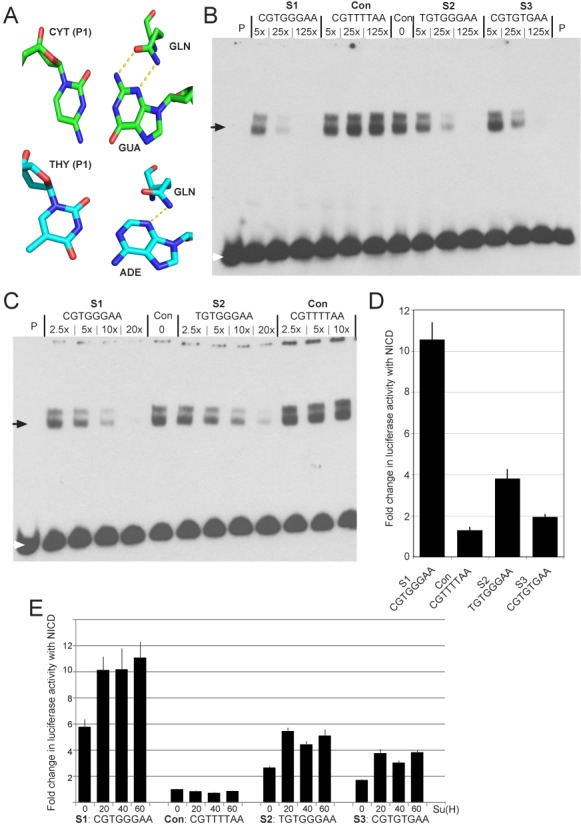
Preference for cytosine over thymine in position 1 of the CSL DNA consensus sequence. (**A**) Physico-chemical explanation for the preference of cytosine over thymine in position 1: the complementary guanine can offer two functional groups for making hydrogen-bond contacts (NH_2_ and aromatic N), while the adenine can only offer one functional group (aromatic N) for an interaction with glutamine (GLN: residue 222 in murine CSL, residue 401 in Lag-1). (**B**) EMSA measuring CSL binding in the presence of different concentrations of the indicated competitor oligonucleotides. Con: no competitor, P: probe only. The white arrow indicates the position of unbound probe and the black arrow indicates the position of bound probe in CSL complexes. (**C**) EMSA measuring CSL binding in the presence of lower dilutions of S1 and S2 competitors as indicated, labeled as in B. (**D**) Response of reporters containing the indicated oligonucleotides to NICD, measured as the fold change in luciferase activity relative to controls with pMT in extracts from transfected cells. Activities were normalized to co-transfected renilla plasmid to control for transfection efficiencies. Error bars depict the standard error of the mean from >3 biological replicate experiments. (**E**) Response of reporters containing the indicated oligonucleotides to NICD, in the presence of varying concentrations of additional CSL as indicated. Fold change in luciferase activity was measured as in D. Error bars depict the standard error of the mean from >3 biological replicate experiments.

To ascertain whether these features only emerged when motifs with low predicted binding energies were included, the nucleotide frequencies were plotted for different thresholds. This revealed that the characteristics of C preference at position 1 and sequence tolerance at positions 2 and 6 were evident even when only the motifs with the top 15–20 energies were considered (Figure 1D). We therefore further scrutinized the FOLDX predictions to investigate the strong cytosine bias at position 1 (Table 1). The energetic distribution shows that FOLDX penalizes the presence of a thymine in position 1 by ca. 2 kcal/mol*.* This difference can be mainly attributed to the H-bond energy between side chains and could be explained by the contacts made by a glutamine residue (222 in murine CSL) within the BTD of CSL. This glutamine residue can make hydrogen bond contacts with the complementary base in position 1; guanine when position 1 is a cytosine, adenine when position 1 is a thymine (Figure 2A). The guanine can make a bidentate interaction (NH_2_ and aromatic N) with the glutamine, while the adenine can only present one group (aromatic N) to hydrogen bond with the glutamine, providing a physico-chemical explanation for the preference of cytosine over thymine in position 1. Indeed, the single X-ray structure where CSL is bound to a DNA motif with cytosine in position 1 (PDB ID 3IAG, -CGTGTGAA-, (44)) shows such a bidentate interaction between the glutamine and guanine, confirming that this contact occurs.

**Table 1.** FOLDX analysis of energetics: difference between thymine and cytosine in position 1 of the DNA binding sequence (energy expressed in kcal/mol)

DNA sequence	Clash DNA	Clash protein	Interaction energy	Sidechain H-bond
CGTGGGAA	11.16	37.67	−12.94	−16.75
TGTGGGAA	11.36	37.74	−10.93	−14.76

To exclude the possibility that the preference for cytosine in position 1 was a consequence of specific elements within the 3BRG structure, we similarly investigated two other CSL–DNA structures with FOLDX. Firstly, using the structure of mouse CSL bound to a slightly different DNA sequence CGTGTGAA (PDB ID: 3IAG) (44), we found a similar preference for cytosine in position 1 (Supplementary Table S5). Secondly, the FOLDX calculation using the Lag-1 (the CSL homologue in *Caenorhabditis elegans*) structure (PDB ID 1TTU) bound to the consensus DNA sequence TGTGGGAA (15) also yielded a preference for cytosine in position 1 (Supplementary Table S5). However, in this case, the difference between cytosine and thymine at position 1 was smaller than for the two mouse CSL–DNA structures analyzed. Nevertheless, the additional calculations suggest that the preference for cytosine over thymine at position 1 is a feature conserved for all CSL orthologs, which is consistent with previous studies demonstrating that CSL from mouse, *Drosophila* and worm all have similar binding characteristics (44).

### Functional relevance of FOLDX predictions: cytosine at position 1

The FOLDX results suggest that CSL motifs with C at position 1 will have higher binding energies. A preference for cytosine in position 1 is not represented in the canonical PWM but was indicated by results from protein binding microarrays (PBM; (19)) and by bacterial 1-hybrid experiments testing Lag-1 binding-site specificities (50).

To investigate CSL binding characteristics further, we compared the C/T motif variants in two assays. First, we tested the ability of the C (S1) and T (S2) variants to compete for binding using an electro-mobility shift assay (EMSA). In these experiments, binding of *Drosophila* CSL to labeled S1 sequence in the presence of differing concentrations of unlabeled S1 and S2 was measured. For comparison, we used a variant with a T at position 5 (CGTGTGAA; S3), because this motif is not often represented in sequences contributing to the PWM. While both S1 and S2 were able to out-compete most of the labeled sequence when present in 25× excess, there was a marked (3.67-fold) difference between the two (Figure 2B, Supplementary Figure S2). Similar differences were also evident at lower dilutions (e.g. 2.84-fold difference at 10× excess; Figure 2C). The S3 sequence competed less well than S2, exhibiting a 9-fold difference with S1 and a 2.4-fold difference with S2 when present at 25× excess (Figure 2B).

Second, the activity of luciferase reporters containing four copies of each motif (arranged as two paired sites) was measured (Figure 2D). Again the S2, T variant, was considerably less active than the S1, C variant, exhibiting 36% of the luciferase signal, although it performed significantly better than the S3 sequence. Furthermore, the differences in the activities of the S1 and S2 sequences were not ameliorated by increasing the amount of CSL over the endogenous levels (Figure 2E). All these data therefore support the notion that sequences containing C at position 1 have stronger binding/function than those with T. Nevertheless, it is evident that the S2 motif with T at position 1 still retains significant binding, contrary to the FOLDX predictions.

**Figure 3.**
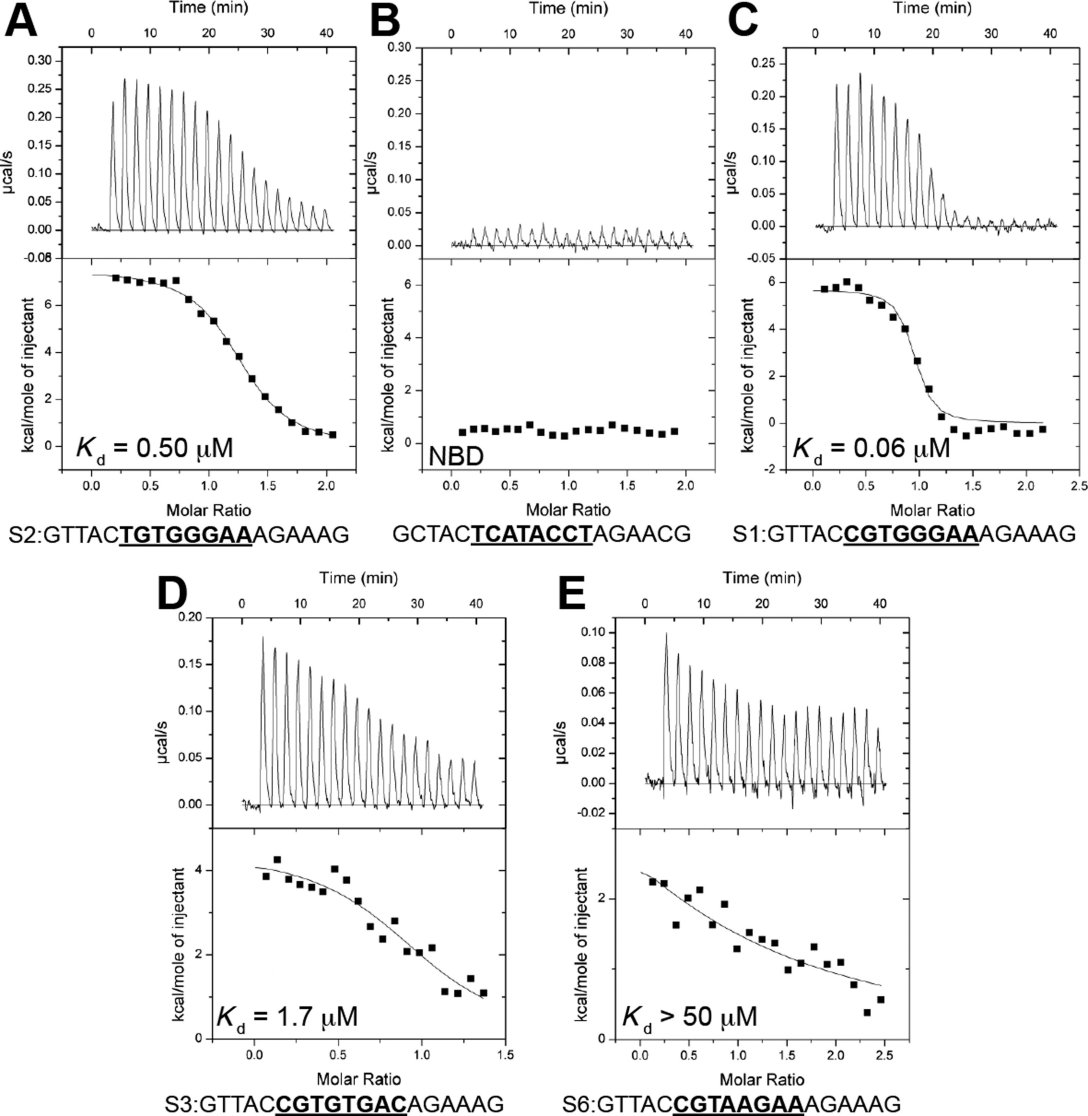
CSL–DNA ITC binding experiments. (**A–E**) Figure shows representative thermograms (raw heat signal and nonlinear least-squares fit to the integrated data) for CSL binding to different DNA duplexes. Relative affinities and specific DNA sequences are shown for each experiment. Twenty titrations were performed per experiment, consisting of 14 μl injections that were spaced 120 s apart.

As the functional studies support the notion that the C at position 1 is more favorable for CSL activity, we subsequently measured the binding affinities of CSL for the C/T variants by ITC (Figure 3). Using purified murine CSL protein with chemically synthesized oligomeric DNA duplexes, the energetics of binding to C and T variant forms of the consensus motif was measured. The results clearly demonstrated that CSL binds more strongly to the sequence with C at position 1, exhibiting a >8-fold difference in the calculated dissociation constant (*K*_d_). Thus, C at position 1, which is highlighted by the FOLDX predictions, does make a significant contribution to the binding preference of CSL.

### Functional relevance of FOLDX predictions: tolerance at positions 2 and 6

**Figure 4.**
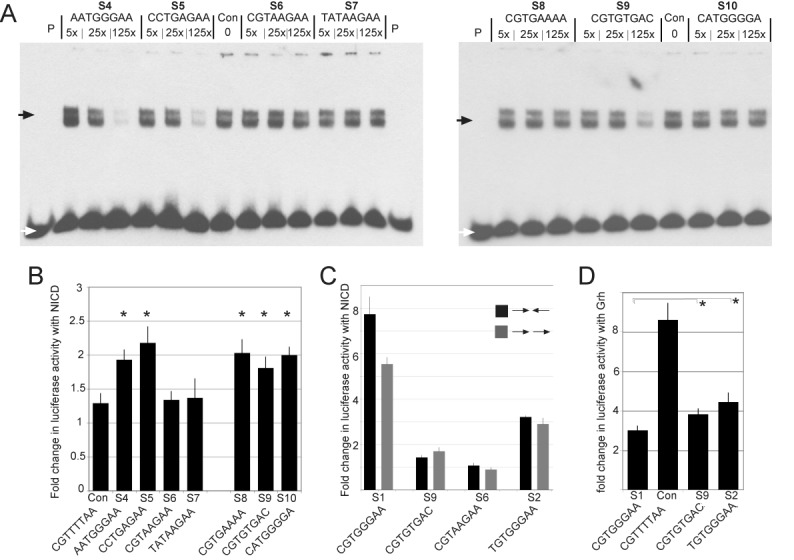
Functional relevance of FOLDX predictions. (**A**) EMSA measuring CSL binding in the presence of different concentrations of the indicated competitor oligonucleotides. Con: no competitor, P: probe only. White arrow indicates the position of unbound probe and black arrow indicates the position of bound probe in CSL complexes. (**B**) Response of reporters containing the indicated oligonucleotides to NICD, measured as the fold change in luciferase activity in extracts from transfected cells. Activities were normalized to co-transfected renilla plasmid to control for transfection efficiencies. Error bars depict the standard error of the mean from >3 biological replicates. * indicates that the response was significantly different from the control (*P* < 0.05, paired t-test). (**C**) Assays were as in B, reporters contained indicated oligonucleotides in palindromic (SPS, black) or parallel (gray) orientations. The difference between the SPS and parallel orientations was not significant (*P* = 0.053; paired t-test). (**D**) Response of reporters containing the indicated oligonucleotides to Grh, measured as the fold change in luciferase activity. Lower levels of activity indicate that the oligonucleotide confers repression by CSL. S1, S9 and S2 were all significantly different from the control (*P* < 0.01, paired t-test); * indicates that repression from S1 was significantly different from S2 (*P* < 0.01) and from S9 (*P* < 0.05).

The FOLDX predictions suggest that, over the range of the top 220 motifs, there is no strong sequence preference at position 2. Canonical CSL sequence logos indicate that guanine is preferred at this position, although variations with high scores are found in both the PBM dataset of CSL bound DNA sequences (19) and are represented in MotifMap/TRANSFAC-derived CSL PWMs. For example the sequence AATGGGAA (S4) scored highly in PBM (19) and is in the top 1% of motifs predicted by the PWM (e.g. using Patser (51)). This motif is also present at quite high frequency in regions occupied by CSL in ChIP (fourth most enriched motif, 5.19% peaks contain this motif) and functional CATG(G/A)GAA motifs are present in several CSL regulated enhancers (52). Another FOLDX variant, CCTGAGAA (S5), was above the threshold of binding in PBM analysis (19) and included as a contributor to some canonical PWM. Comparing the activity of these two sequences in the EMSA competition assays, we found that both had intermediate capability to compete with the canonical sequence (detectable at 125× excess; Figure 4A). Likewise, both demonstrated functional activity in the reporter assays, exhibiting >2× stimulation in the presence of NICD, indicating the potential for variations at this position (Figure 4B). Although the activities were low, they nevertheless were significantly different from control sequences where all critical residues were replaced by T (CGTTTTAA: Con) and from a motif where positions 4/5 were substituted (CGTAAGAA; S6); Figure 4B. Surprisingly, the latter was previously reported to have stronger binding than the S1, TGTGGGAA position 1 variant (53). The ITC analysis confirms the low affinity binding for CGTAAGAA (*K*_d_ of ∼50 μM; Figure 3F) although, notably, this is still significantly better than detected for a control ‘unbound’ DNA sequence (TCATACCT; Figure 3C). Finally, a related sequence (TATAAGAA; S7), which was previously reported to be bound by CSL (Su(H)) (54), failed to compete at 125× in the EMSA experiments and showed no response to NICD in the reporter assays (Figure 4A and B).

FOLDX also predicts that adenine at position 6 would be energetically favourable, although conventionally only guanine is considered at this position, with CGTGAAAA (S8) one of its top ranked motifs. In EMSA experiments the S8 sequence exhibited very weak competition, but nevertheless its activity was similar to the position 2 variant motifs in the luciferase experiments (Figure 4B), and was considerably better than sequences with position 4/5 substitutions. Furthermore, in ITC assays there was measurable, albeit weak, binding to CSL (*K*_d_ >50 μM). Such CGTGAAAA motifs may therefore have some functional binding *in vivo*, although the data do not support their high energetic ranking by FOLDX.

### Relevance of other motif variants

In addition, we tested several other sequences that fell within the top 1% of FOLDX predictions, including several that had been identified as binding/functional by previous assays. For example, it has previously been shown how mutations at positions 5 (6) and 8 (45) of the DNA sequence recognized by CSL can be tolerated, mainly due to a reduced number of specific side chain–nitrogen base interactions (15). For this reason, the sequence CGTGTGAC (S9) has been tested using ITC, showing that it is bound by CSL with a moderate *K*_d_ (1.7 μM; Figure 3E), which is significantly better than the CGTAAGAA (S5) motif. In agreement, this sequence exhibited intermediate levels of binding in the EMSA competition assay and gave rise to ∼2-fold levels of activation in response to NICD, similar to other motifs with intermediate binding (Figure 4A and B).

A second sequence in the top 1% of FOLDX motifs, but not predicted based on MotifMap/TRANSFAC-derived PWMs nor classified as bound in the PBM analysis (19), is CATGGGGA (S10). This exhibited similar low levels of activity in response to NICD in the luciferase assays, although the ability to compete for binding in EMSA was not detectable at 125× excess (Figure 4A and B). Finally, CTGGGGAA was ranked 85th on the basis of FOLDX predicted binding energies, but was inactive in the reporter assays and failed to compete even at 500× excess in EMSA assays (data not shown). Thus, it appears that only some of the structure-based predictions are informative about potentially relevant site variants.

### Relevance of sequence orientation and contribution to repression

Finally, we tested whether there was a significant difference in the activity of sequence motifs depending on their orientation and on whether motifs behaved similarly in repression assays. Previous experiments have demonstrated that so-called paired sites (SPS), where two CSL motifs are arranged palindromically, often exhibit greater response to NICD because they favor dimerization between NICD molecules in adjacent complexes (55). It is possible therefore that some less favored motifs may exhibit activity when in the SPS configuration, because of the added stabilization from NICD:NICD interactions. Such sequences might function less well when in a head-to-tail orientation and/or in conferring repression.

To determine whether the DNA orientation affects the transcriptional activation from the weak sites, we re-tested different DNA sequences in parallel orientations for their ability to respond to NICD (Figure 4C). Under these conditions, most sequences gave rise to similar activity whether in SPS or in parallel arrangements. Only the strong S1 consensus site with C at position 1 showed a slight difference in activity, conferring 1.5× higher activity in the SPS orientation (Figure 4C; *P* = 0.053). Strikingly, the T (S2) variant did not exhibit such a difference nor did the others tested (S5, S8, S7; Figure 4C). Thus, the sequence arrangements do not appear to account for the ability of ‘weaker’ sequences to confer transcriptional activation; under these conditions of high NICD expression the CSL-mediated regulation is largely unaffected by the orientation of the motifs (although it should be noted that these experiments do not address the importance of NICD dimerization *per se*).

To further explore the activity of different CSL binding motifs, they were tested in a repression assay. The reporters also contain binding sites for the *Drosophila* transcriptional activator Grainyhead (Grh). The ability of different sequence motifs to confer CSL-mediated inhibition of Grh can therefore be assessed based on the expression levels in the presence of Grh: effective repression is evident as a reduction in the expression levels, comparing the effects of different CSL motifs with a control (CGTTTTAA) sequence (Figure 4D). As for activation, the most robust repression was detected with reporters containing CGTGGGAA (S1). Substitution of T at position 1 (S2) resulted in a decrease in the magnitude of repression, indeed the position 1 variant TGTGGGAA behaved more similarly to the non-canonical sequence CGTGTGAA (S3). Overall, it appears that the motif requirements for CSL-mediated repression are similar to those for activation with NICD, with the position 1 C variant being the most effective.

### MD simulations demonstrate effects of different DNA sequences on internal CSL dynamics

The EMSA and reporter assays indicate that, although CSL has a strong preference for the two consensus sequences, its activity is sensitive to the base at position 1, with preference for a C. Furthermore, probing a range of sequences has revealed that a broad repertoire of motifs can be bound by CSL (e.g. Figures 3 and 4), with many having quite similar functional activities. These subtle differences in binding versus functional activities prompted us to investigate the effects of different DNA sequences on CSL dynamical behavior, by performing MD simulations of CSL in the presence of four different DNA sequences.

Simulations of the two DNA ‘consensus’ sequences, CGTGGGAA (S1) and TGTGGGAA (S2), were compared to unbound CSL and to two mutated DNA sequences: CGTGTGAC (S9), which exhibits intermediate binding and transcriptional regulation, and CGTAAGAA (S6), which exhibits little/no binding or activity but has a *K*_d_ significantly different from negative controls (Table 2, Figure 3C, E, F).

**Table 2.** Calorimetric data for various DNA sequences binding to CSL

Ligand (syringe)	*K* (M^−1^)	*K*_d_ (μM)	Δ*G*° (kcal/mol)	Δ*H*° (kcal/mol)	−*T*Δ*S*° (kcal/mol)
CGTGGGAA	2.2 ± 1.4×10^7^	0.06	−9.4 ± 0.3	5.1 ± 1.0	−14.5 ± 0.7
TGTGGGAA	2.0 ± 0.5×10^6^	0.50	−8.2 ± 0.1	8.8 ± 1.2	−17.0 ± 1.0
CGTGTGAC	5.9 ± 1.0×10^5^	1.72	−7.5 ± 0.1	4.8 ± 1.0	−12.3 ± 1.0
CGTAAGAA	—	>50	—	—	—
TCATACCT*	NBD	NBD	NBD	NBD	NBD

*Negative control.

All experiments were performed at 10°C. Values are the mean of at least three independent experiments and errors represent the standard deviations of multiple experiments.

NBD = no binding detected.

**Figure 5.**
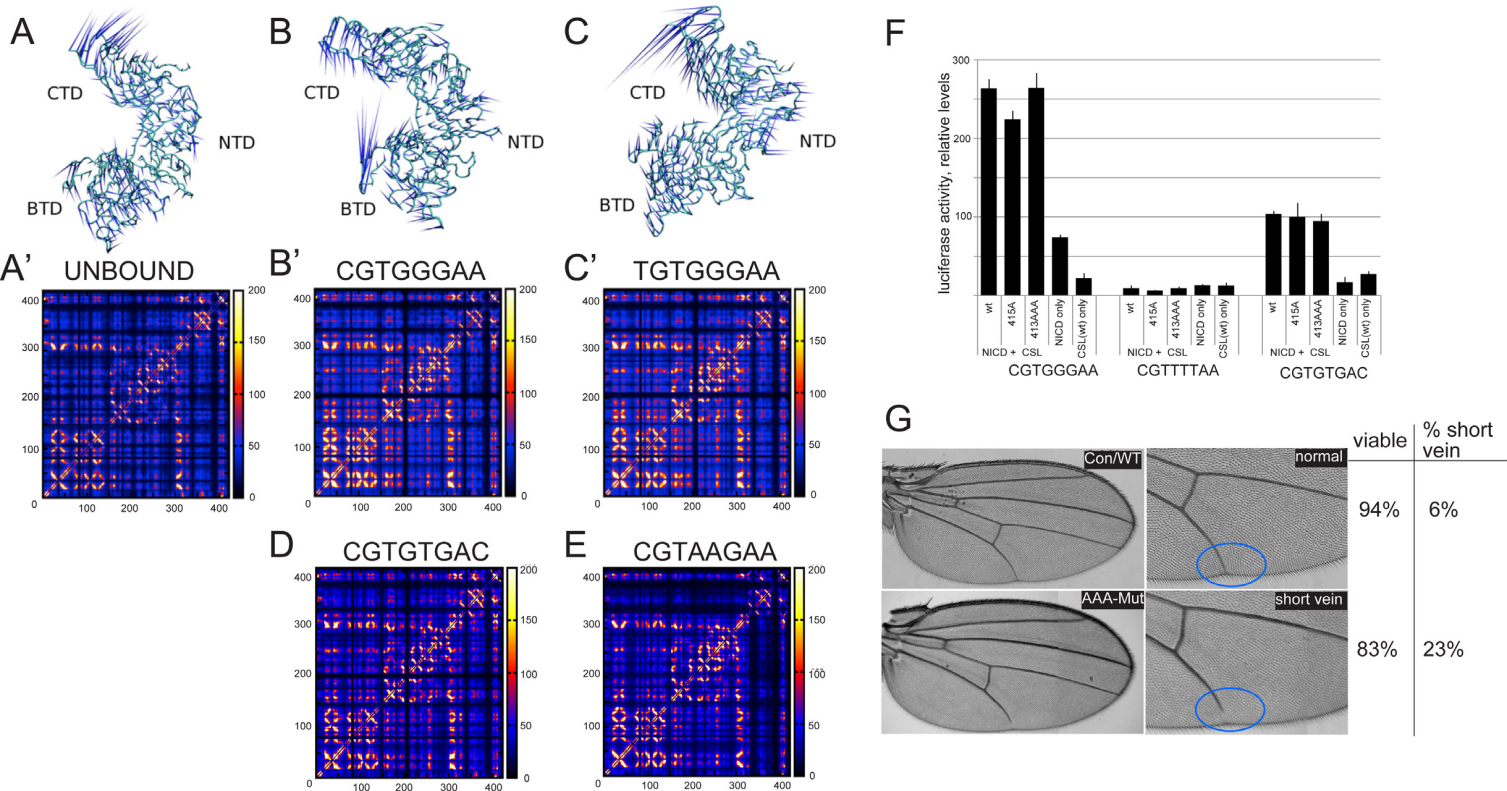
Effects of DNA sequences on internal CSL dynamics and functional implications. (**A–E**) Results from MD simulations showing inter-domain correlations when CSL is bound to different sequences as indicated. In particular, A–C show the comparison between the projection of the first eigenvector on the CSL structure, in the presence or absence of DNA sequences. The spikes of the porcupine plots indicate the principal motions (i.e. the motion described by the first eigenvector) for each C-alpha, while the length indicates the intensity of the motion. A’–C’ and D–E show a comparison of the ICRM matrices between the different systems studied. (F) Consequences of mutations in CSL on activity of reporter genes containing the indicated sequences. Unmutated CSL (WT), single mutant CSL (415A) and triple mutant CSL (413AAA) were co-transfected with NICD (ratio of DNAs was 1:5) and the expression of the indicated reporter measured relative to co-transfected renilla control. Error bars depict the standard error of the mean from >3 biological replicates. (G) Adult wings from flies with either wild-type or mutated CSL rescue plasmids, right panel is a higher magnification of the region with altered venation (blue circles). Tables indicate the proportion of flies that were viable in each case, and the proportion of viable flies whose wings had venation defects. *N* > 200.

As the RMSD and RMSF analyses (Supplementary Figures S3 and S4) did not show large differences between the complexes with the four test sequences, the intra-domain correlation of the complexes and CSL in its unbound state was calculated using the ICRM. This is a widely used tool to study dynamical differences between protein–DNA, protein–protein and protein–small molecules interactions (32,37,56). The results reveal that both ‘consensus’ complexes produce a similar intra-domain correlation (Figure 5B and C), as is also confirmed by the calculation (Table 3) and by the direction and the intensity of the principal motions made by the CSL residues during the simulations (shown by the projection of the eigenvalue corresponding to the first eigenvector on the structure; Figure 5B’ and C’). This intra-domain correlation is not seen in the equivalent matrix from unbound CSL, which exhibits different (relative) domain–domain movements (Figure 5A) and strongly reduced domain–domain correlations (Figure 5A’, Table 3). These results imply that the bound DNA configures the system such that the domains within CSL are strongly correlated. Furthermore, the internal correlation of CSL is reduced in proportion to the binding affinity (Table 3). Interestingly, the two mutated sequences analyzed, CGTGTGAC (S9) and CGTAAGAA (S6), gave rise to different behaviors (Figure 5D and E). With the intermediate functional motif, CGTGTGAC, some internal correlations were retained, producing a profile that is intermediate between the consensus sequences and the unbound state (Figure 5D). With the non-functional motif, CGTAAGAA, very limited internal correlation remained, generating a profile similar to CSL in its unbound state (Figure 5E).

**Table 3.** Ranked eigenvalues from the ICRM matrices of the complexes studied

Complex	First eigenvalue
TGTGGGAA	23 938
CGTGGGAA	23 716
CGTGTGAC	20 934
CGTAAGAA	19 073
UNBOUND	17 821
413AAA	16 100

The observation that the internal correlation of CSL is reduced in proportion to the binding affinity may be one factor that helps in understanding how CSL selects different DNA sequences. In addition, if the internal dynamics change depending on the sequence, this might impact on subsequent interactions between CSL and other proteins. In order to test possible impacts of such internal dynamics, we assessed the consequences of mutating three residues (413AAA) that contribute to the terminal part of the beta strand, which links the BTD domain with the CTD domain (Figure 1B). MD simulations indicated that such a mutated protein has reduced domain–domain interactions (Table 3). However, the ability of the 413AAA mutant to stimulate transcription in the presence of NICD was similar to that of the native protein in the context of both CGTGGGAA and CGTGTGAC reporter constructs (Figure 5F). Likewise, mutation of a single residue (A415) had no impact on CSL activity in these assays. Finally, the consequence of these mutations on the function of CSL *in vivo* was assessed in transgenic rescue experiments (Figure 5G). A genomic fragment expressing the wild-type protein is able to fully rescue flies with loss of endogenous CSL function (*Su(H)^SF8^/Su(H)^AR9^* transheterozygote, Figure 5G). In contrast, a similar fragment carrying the 413AAA mutated version shows reduced function, viability is compromised and the surviving flies have wing venation defects (Figure 5G). Although this suggests that the protein may function less effectively when the inter-domain protein dynamics are perturbed, it is also possible that the 413AAA mutation perturbs other aspects of CSL function.

## DISCUSSION

One of the challenges in understanding how TFs regulate genes resides in our limited ability to predict where they will bind in the genome. Even taking into consideration the numerous regulatory layers that influence the accessibility of binding sites in chromatin, TFs are frequently found to occupy different sites from those predicted. One reason for this disparity is the extent of knowledge about the full spectrum of recognition motifs. For example, PWM libraries are often biased due to the historical manner in which many were constructed, based around the first known motifs for a given TF (57). Furthermore, classic PWM-based approaches treat all nucleotides along the sequence motif independently and cannot utilize information that arises from correlation analysis (58). Confronted by these challenges, our strategy was to use computational modeling, based on protein structural properties, to probe the specificity of CSL binding. In doing so we have clarified important features. For example positions that were thought to be biased toward a specific nucleotide (positions 2 and 6), as illustrated by MotifMap/TRANSFAC PWMs, were predicted by the modeling to accommodate a wider spectrum of nucleotides. Some of these differences, notably the variability at position 2, were also detected by PBM analysis (19) and motifs with these variations were demonstrably functional in our *in vitro* binding and reporter assays.

Conversely, the modeling predicted a strong preference at position 1, which was quantified experimentally. Thus, motifs with a C at position 1 performed consistently better than those with T. Together, the results demonstrate that computational modeling from the crystal structure can expand the knowledge about functional target sites, even in cases of otherwise well-characterized TFs such as CSL. However, it is evident that the computational predictions also have biases. For example variations at position 1 were energetically penalized. As a consequence, the results are best used in combination with other data rather than as a predictive tool on their own. One possible reason may be related to the fact that the FOLDX calculation is based on the assumption that CSL binds with a similar conformation to each and every DNA sequence, while there is evidence to suggest that TFs can slightly change their conformation when bound to high or low affinity DNA sequences (59).

One important question is how TFs select the correct binding site amongst others that are very close energetically. Indeed, there is a notion that many of the lower affinity interactions between TFs and DNA primarily represent a buffering mechanism to retain those molecules close to the DNA, while only a few binding events play an actual regulatory role (60). On the atomic level there must be mechanisms to discern these different forms of binding events. To investigate whether the specific DNA sequence present could have an impact on the way the protein behaves, we used MD simulations, to determine the influence of bound DNA on the ability of CSL to transmit a dynamic signal within its structure. Our results predict a profound effect of DNA binding on the inter-domain correlated motions, with lower affinity sequences demonstrating a reduced correlation compared to high affinity sites. For example, although the structural modeling suggests that compensatory interactions can occur when specific DNA contacts are lost, such as in the CSL complexes with either CGTGTGAC or CGTAAGAA, nevertheless these interactions do not give rise to the same long-range domain–domain communication.

By revealing that different DNA sequences can propagate different dynamic signals through the protein, this approach suggests the possibility of an emergent behavior that transduces a dynamic signal modulating gene expression. The inter-domain signaling within CSL that is elicited by DNA binding could thus be important to distinguish functional from non-functional DNA interaction sites and could in turn affect the recruitment of other factors to the bound TF. Indeed, such allosteric changes in CSL have been proposed to affect the formation of the tertiary complex with NICD based on other modeling strategies (61). Furthermore, DNA-induced allosteric changes in TFs have been proposed to play important roles in transcriptional regulation (62). Thus, depending on the protein–DNA binding event, different communication regimes could be generated and influence the motion and the energy landscape within the protein to modulate its interactions. Despite these intriguing models, mutations in residues that should perturb the inter-domain correlations have at best modest consequences for the function of CSL in the assays used. Thus, no specific differences were detected in CSL's ability to stimulate transcription from different sequences when the domain–domain communications were compromised under conditions with high levels of expressed proteins. However, such a mutated protein did have reduced function *in vivo*, which is consistent with the inter-domain communication being important for full activity of CSL under physiological conditions, although there are also other possible explanations.

In summary, *in silico* approaches to investigate the mechanisms of CSL binding have revealed additional features, increasing our understanding of the repertoire of sequences that may be functional. Furthermore, the results suggest that the specific sequence bound may in turn impact on the outcome of the binding event, although our experiments could not confirm a direct effect on transcriptional outcome. Still, such dynamics may be important for the functional TF binding to be distinguished from non-functional, by yet unidentified factors.

## SUPPLEMENTARY DATA

Supplementary Data are available at NAR Online.

SUPPLEMENTARY DATA
