# Supplementary material for: A combination of computational and experimental approaches identifies DNA sequence constraints associated with target site binding specificity of the transcription factor CSL
Source: Nucleic Acids Res. 2014 Aug 11;42(16):10550–63. doi: 10.1093/nar/gku730 (PMC4176381; doi:10.1093/nar/gku730)
Supplement: SUPPLEMENTARY DATA [file supp_42_16_10550__index.html]

A combination of computational and experimental approaches identifies DNA sequence constraints associated with target site binding specificity of the transcription factor CSL — A combination of computational and experimental approaches identifies DNA sequence constraints associated with target site binding specificity of the transcription factor CSL — SUPPLEMENTARY DATA 

# A combination of computational and experimental approaches identifies DNA sequence constraints associated with target site binding specificity of the transcription factor CSL

## SUPPLEMENTARY DATA

**Files in this Data Supplement:**

- SUPPLEMENTARY DATA
