# Supplementary material for: A combination of computational and experimental approaches identifies DNA sequence constraints associated with target site binding specificity of the transcription factor CSL
Source: Nucleic Acids Res. 2014 Aug 11;42(16):10550–63. doi: 10.1093/nar/gku730 (PMC4176381; doi:10.1093/nar/gku730)
Supplement: SUPPLEMENTARY DATA [file supp_gku730_nar-00973-z-2014-File007.pdf]

Supplementary Figures and Tables

Table S1. Oligonucleotides used in EMSA.

	Sense	Antisense
M1	ACCGAAAC**CGTGGGAA**CTGGTAGAAAG	CTTTCTACCAG**TTCCCACG**GTTTCGGT
M2	ACCGAAAC**CGTGCCTA**CTGGTAGAAAG	CTTTCTACCAG**TAGGCACG**GTTTCGGT
M3	ACCGAAAC**TGTGGGAA**CTGGTAGAAAG	CTTTCTACCAG**TTCCCACT**GTTTCGGT
M4	ACCGAAAC**CGTGTGAA**CTGGTAGAAAG	CTTTCTACCAG**TTCACACG**GTTTCGGT
M5	ACCGAAAC**AATGGGAA**CTGGTAGAAAG	CTTTCTACCAG**TTCCCATT**GTTTCGGT
M6	ACCGAAAC**CCTGAGAA**CTGGTAGAAAG	CTTTCTACCAG**TTCTCAGG**GTTTCGGT
M7	ACCGAAAC**CGTAAGAA**CTGGTAGAAAG	CTTTCTACCAG**TTCTTACG**GTTTCGGT
M8	ACCGAAAC**TATAAGAA**CTGGTAGAAAG	CTTTCTACCAG**TTCTTATA**GTTTCGGT
M9	ACCGAAAC**CGTGAAAA**CTGGTAGAAAG	CTTTCTACCAG**TTTTCACG**GTTTCGGT
M10	ACCGAAAC**CGTGTGAC**CTGGTAGAAAG	CTTTCTACCAG**GTCACACG**GTTTCGGT
M11	ACCGAAAC**CATGGGGA**CTGGTAGAAAG	CTTTCTACCAG**TCCCCATG**GTTTCGGT

Table S2. Primers used to generate different Su(H) luciferase vectors.

Fragments were inserted using XhoI and EcoRI sites.

**Primers used to generate SPS (so-called paired sites).**	
CGTGGGAA	CTATCTCGAGG**CGTGGGAA**CCGAGCTGAAAGTAAGT**TTCCCACG**GGAATCTGACCTAGGTAGTGTGG
	CGATGAATTCTTG**CGTGGGAA**ACTTACTTTCAGCTCGG**TTCCCACG**CCACACTACCTAGGTCAGATTCC
CGTTTTAA	CTATCTCGAGG**CGTTTTAA**CCGAGCTGAAAGTAAGT**TTAAAACG**GGAATCTGACCTAGGTAGTGTGG
	CGATGAATTCTTG**CGTTTTAA**ACTTACTTTCAGCTCGG**TTAAAACG**CCACACTACCTAGGTCAGATTCC
CGTGTGAC	CTATCTCGAGG**CGTGTGAC**CCGAGCTGAAAGTAAGT**GTCACACG**GGAATCTGACCTAGGTAGTGTGG
	CGATGAATTCTTG**CGTGTGAC**ACTTACTTTCAGCTCGG**GTCACACG**CCACACTACCTAGGTCAGATTCC
CGTGTGAA	CTATCTCGAGG**CGTGTGAA**CCGAGCTGAAAGTAAGT**TTCACACG**GGAATCTGACCTAGGTAGTGTGG
	CGATGAATTCTTG**CGTGTGAA**ACTTACTTTCAGCTCGG**TTCACACG**CCACACTACCTAGGTCAGATTCC
CGTAAGAA	CTATCTCGAGG**CGTAAGAA**CCGAGCTGAAAGTAAGT**TTCTCACG**GGAATCTGACCTAGGTAGTGTGG
	CGATGAATTCTTG**CGTAAGAA**ACTTACTTTCAGCTCGG**TTCTCACG**CCACACTACCTAGGTCAGATTCC
CGTGAAAA	CTATCTCGAGG**CGTGAAAA**CCGAGCTGAAAGTAAGT**TTTTCACG**GGAATCTGACCTAGGTAGTGTGG
	CGATGAATTCTTG**CGTGAAAA**ACTTACTTTCAGCTCGG**TTTTCACG**CCACACTACCTAGGTCAGATTCC
CATGGGGA	CTATCTCGAGG**CATGGGGA**CCGAGCTGAAAGTAAGT**TCCCCATG**GGAATCTGACCTAGGTAGTGTGG
	CGATGAATTCTTG**CATGGGGA**ACTTACTTTCAGCTCGG**TCCCCATG**CCACACTACCTAGGTCAGATTCC
CCTGAGAA	CTATCTCGAGG**CCTGAGAA**CCGAGCTGAAAGTAAGT**TTCTCAGG**GGAATCTGACCTAGGTAGTGTGG
	CGATGAATTCTTG**CCTGAGAA**ACTTACTTTCAGCTCGG**TTCTCAGG**CCACACTACCTAGGTCAGATTCC

TATAAGAA	CTATCTCGAGG**TATAAGAA**CCGAGCTGAAAGTAAGT**TTCTTATA**GGAATCTGACCTAGGTAGTGTGG
	CGATGAATTCTTG**TATAAGAA**ACTTACTTTCAGCTCGG**TTCTTATA**CCACACTACCTAGGTCAGATTCC
TGTGGGAA	CTATCTCGAGG**TGTGGGAA**CCGAGCTGAAAGTAAGT**TTCCCACA**GGAATCTGACCTAGGTAGTGTGG
	CGATGAATTCTTG**TGTGGGAA**ACTTACTTTCAGCTCGG**TTCCCACA**CCACACTACCTAGGTCAGATTCC
AATGGGAA	CTATCTCGAGG**AATGGGAA**CCGAGCTGAAAGTAAGT**TTCCCATT**GGAATCTGACCTAGGTAGTGTGG
	CGATGAATTCTTG**AATGGGAA**ACTTACTTTCAGCTCGG**TTCCCATT**CCACACTACCTAGGTCAGATTCC
CTGGGGAA	CTATCTCGAGG**CTGGGGAA**CCGAGCTGAAAGTAAGT**TTCCCCAG**GGAATCTGACCTAGGTAGTGTGG
	CGATGAATTCTTG**CTGGGGAA**ACTTACTTTCAGCTCGG**TTCCCCAG**CCACACTACCTAGGTCAGATTCC
**Primers used to generate Su(H) motifs in parallel orientations.**	
CGTGGGAA	CTATCTCGAGG**CGTGGGAA**CCGAGCTGAAAGTAAGT**CGTGGGAA**GGAATCTGACCTAGGTAGTGTGG
	CGATGAATTCTTG**TTCCCACG**ACTTACTTTCAGCTCGG**TTCCCACG**CCACACTACCTAGGTCAGATTCC
CGTGTGAC	CTATCTCGAGG**CGTGTGAC**CCGAGCTGAAAGTAAGT**CGTGTGAC**GGAATCTGACCTAGGTAGTGTGG
	CGATGAATTCTTG**GTCACACG**ACTTACTTTCAGCTCGG**GTCACACG**CCACACTACCTAGGTCAGATTCC
CGTAAGAA	CTATCTCGAGG**CGTAAGAA**CCGAGCTGAAAGTAAGT**CGTAAGAA**GGAATCTGACCTAGGTAGTGTGG
	CGATGAATTCTTG**TTCTTACG**ACTTACTTTCAGCTCGG**TTCTTACG**CCACACTACCTAGGTCAGATTCC
TGTGGGAA	CTATCTCGAGG**TGTGGGAA**CCGAGCTGAAAGTAAGT**TGTGGGAA**GGAATCTGACCTAGGTAGTGTGG
	CGATGAATTCTTG**TTCCCACA**ACTTACTTTCAGCTCGG**TTCCCACA**CCACACTACCTAGGTCAGATTCC

Table S3. Luciferase Construct pGL3v3

A BglII site was inserted into pGL3::min plasmid by PCR amplification. Then 3x Grainyhead binding sites were cloned and inserted using MluI/BglII sites upstream of the multiple cloning sites, this forms the pGL3v3 luciferase construct. Primers used are listed below.

Adding BglII site
5’ GGAAGATCTGAATTCGGTACCCTCGAGGAGCGCCCCAGTATAAATAGAGG 3’
5’ GGAAGATCTCCGCGGACGCGTCCTATCGATAGAGAAATGTTCTGGC 3’

Cloning of Grh sites
5’ TTGGCAGATTTTCAGTAGTTGCAG 3’
5’ ATAAGTTCAATGATGTCCAGTGCAG 3’

Table S4. Sequence of primers used for cloning CSL and 413AAA mutant

CSL genomic cloning
5’ cgaggatccCAAGTTAGATATGGCAATGCACCG 3’
5’ cgaggatccACTGCATATCTGTACTGATGACG 3’

CSL 413AAA mutagenesis
5’ CCACCGACAAGGCTGAGTACgccgcagccGAGGGCATGGGTCCTGTGG 3’
5’ CCACAGGACCCATGCCCTCggctgcggcGTACTCAGC CTTGTCGGTGG 3’

Table S5: comparative FOLDX analysis of the cytosine to thymine mutation in position 1, on three different structures (PDB IDs 3BRG, 3IAG and 1TTU)

DNA Sequence	DNA Sequence	FOLDX ∆E
CGTGGGAA – 3BRG	-12.94	/
TGTGGGAA – 3BRG	-10.93	-2.01
CGTGTGAA – 3IAG	-14.41	/
TGTGTGAA – 3IAG	-13.19	-1.29
CGTGGGAA – 1TTU	-16.85	/
TGTGGGAA – 1TTU	-16.25	-0.6

Figure S1: Additional computational analysis of binding preferences

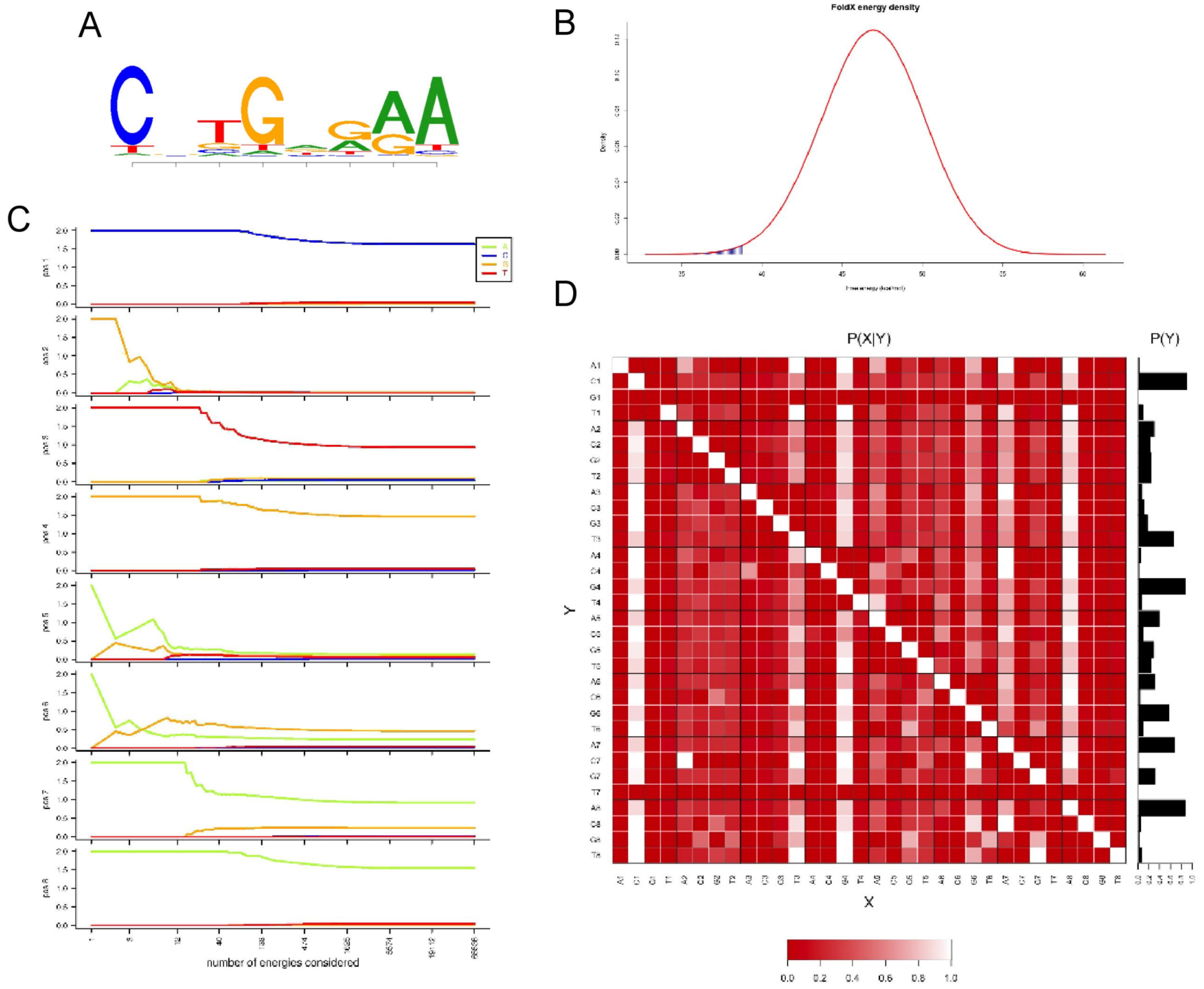

Figure S1: Additional computational analysis of binding preferences

A: Logo compiled from the unweighted FOLDX sequences with energies >3kcal/mol

B: Distribution of FOLDX calculated energies, the region corresponding to the sequences with binding energies >3kcal/mol are shaded

C: Nucleotide preference by position for an increasing number of sequences. Information content for a given nucleotide at each position, sequences have been weighted by their Boltzmann probability.

D: Conditional probability of finding a nucleotide at a certain position, given the nucleotide at another position.

Figure S2: Quantification of EMSA gels in Figure 2B and Figure 2C

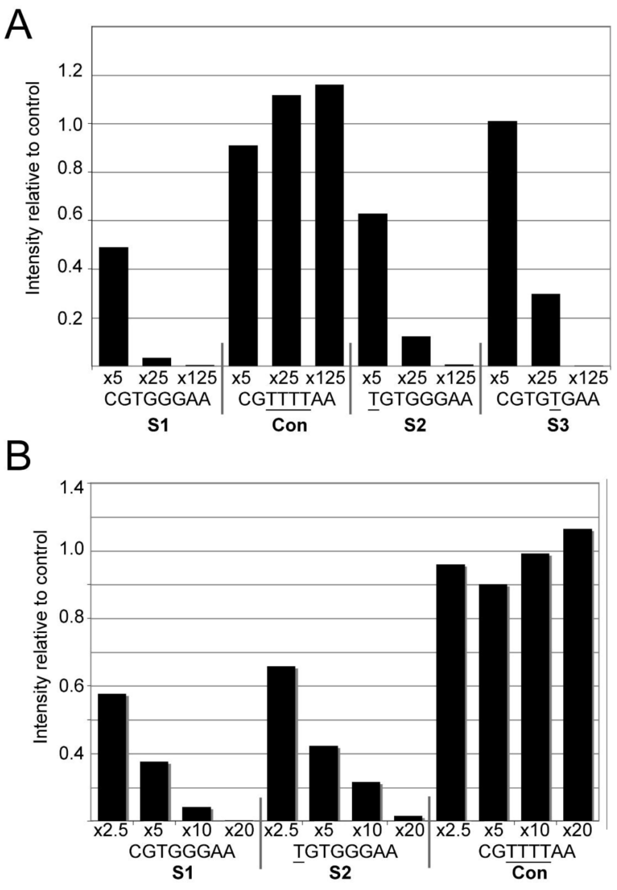

Figure S2: Quantification of bound regions in Fig.2B (A) and Fig. 2C (B) relative to the controls (with no competitor)

Figure S3:

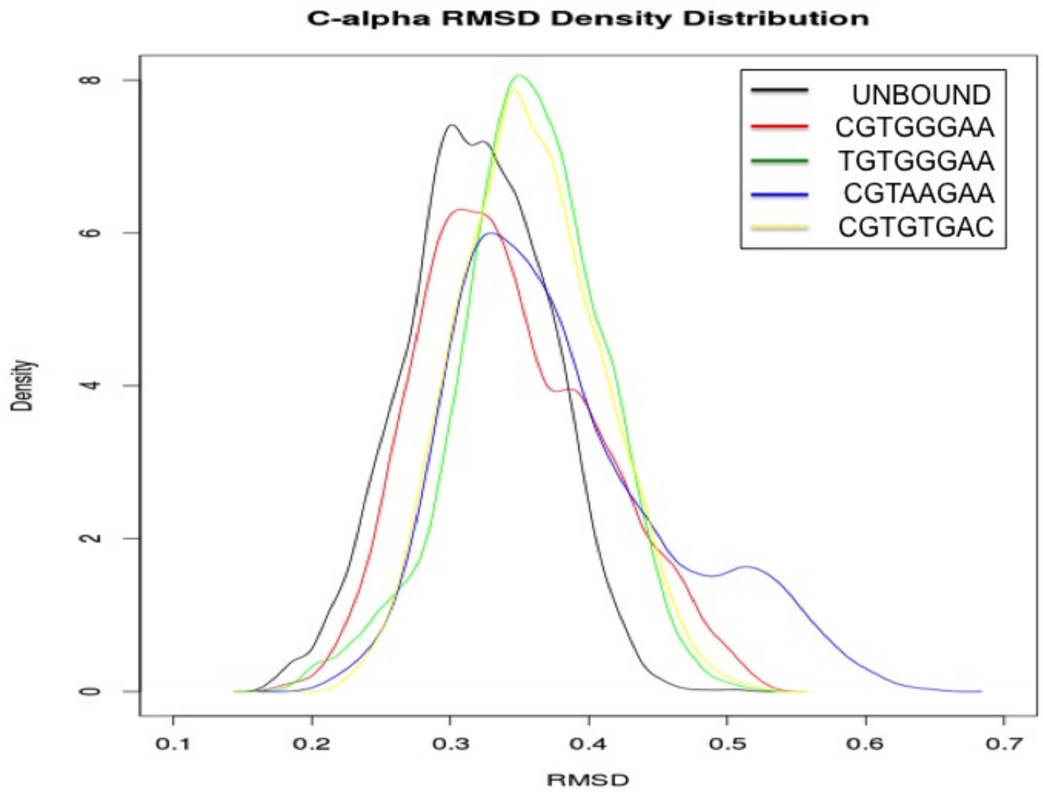

Figure S1: C-alpha RMSD density distribution calculation of the macro-trajectory of 120 ns, for each different system in study (CSL-UNBOUND in black, CGTGGGAA in red, TGTGGGAA in green, CGTAAGAA in blue and CGTGTGAC in yellow).

Figure S4:

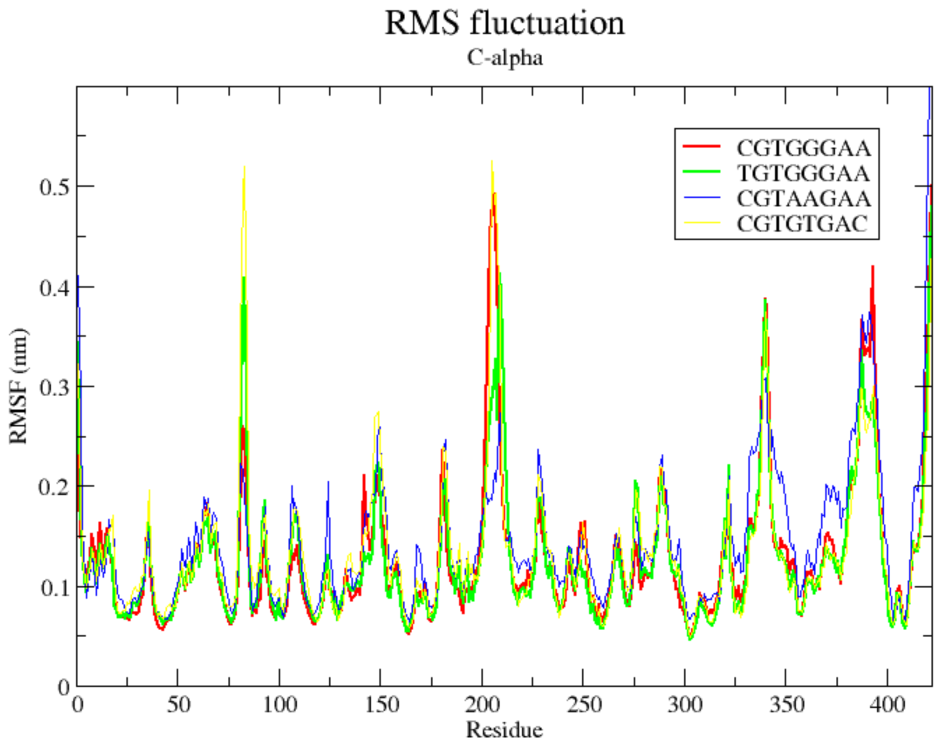

Figure S4: C-alpha RMSF calculation comparison between CSL complexes, bound to four different DNA sequences (CGTGGGAA in red, TGTGGGAA in green, CGTAAGAA in blue and CGTGTGAC in yellow).
